# Psychological inoculation against problematic social media use among adolescents: An experimental study

**DOI:** 10.1111/nyas.70011

**Published:** 2025-07-27

**Authors:** Sameha Alshakhsi, Dena Al‐Thani, Niko Männikkö, Raian Ali

**Affiliations:** ^1^ College of Science and Engineering Hamad Bin Khalifa University Doha Qatar; ^2^ Centre for Research and Innovation Oulu University of Applied Sciences Oulu Finland

**Keywords:** inoculation theory, persuasive design, PSMU, psychological inoculation, social media disorder

## Abstract

This research investigated whether an attitudinal inoculation intervention can build resistance against problematic social media use (PSMU) in adolescents. This experimental study assessed PSMU levels and attitudes toward PSMU before and after the intervention. The intervention utilized scenarios reflecting symptoms of PSMU, including preoccupation, tolerance, withdrawal, persistence, displacement, problem, deception, escape, and conflict. Scenarios employed Cialdini's persuasion principles (reciprocity, liking, social proof, scarcity, authority, and commitment and consistency) and explained these principles to participants. The study included a control group, active inoculation group (participants identified countermeasures to PSMU scenarios), and passive inoculation group (countermeasures were provided). Participants were adolescents aged 11–15 years. A mixed ANCOVA was employed to test the intervention's impact on PSMU, post‐intervention attitude changes, and post‐inoculation talk (PIT) about excessive technology use. Results indicated a significant decrease in PSMU levels following active inoculation, particularly in withdrawal, persistence, displacement, and deception symptoms. The passive inoculation group showed a significant decrease in deception only. No changes were observed in the control group. Regarding PIT, passive inoculation showed a marginally significant increase in negative PIT, whereas active inoculation saw a slight reduction in positive PIT. These findings, while exploratory, suggest attitudinal inoculation's potential for mitigating PSMU and warrant further research.

## Graphical abstract

This study investigated an inoculation‐based intervention to reduce problematic social media use (PSMU) in adolescents. Active inoculation, in which participants generated their own resistance strategies, significantly reduced PSMU symptoms, specifically withdrawal, persistence, displacement, and deception. Passive inoculation, in which participants were provided with resistance strategies, reduced deception only. Changes in post‐inoculation talk about PSMU were also observed.

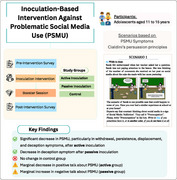

## INTRODUCTION

The rapid advancement of social media technology has fundamentally reshaped the way individuals spend their time, communicate, and portray themselves, particularly among adolescents. According to Pew Research Center, as of 2022, approximately 97% of adolescents use the internet daily, indicating a significant increase from 92% in 2014–15.^1^ Notably, a high percentage of adolescents actively engage with various social media platforms, mostly YouTube (93%), TikTok (63%), Snapchat (60%), and Instagram (59%).^2^ These trends demonstrate how these platforms have become increasingly integrated into the daily lives of adolescents, significantly altering how they interact and consume information. However, with the rise in social media usage, the concerns regarding the excessive and obsessive use of social media have also increased.

Problematic social media use (PSMU), characterized by excessive and obsessive use of social media that interferes with daily activities, can lead to negative consequences, including mental health issues such as depression and anxiety,^3^ low life satisfaction, sleep quality,^4^ academic performance,^5^ cultural and behavioral risks for families,^6^ and even negative psychological outcomes.^7^ Research has indicated that the prevalence of PSMU among adolescents varies, ranging from 4.5% to 25.5%.^8^, ^9^ The increasing prevalence of PSMU and its associated negative impacts highlight the need to find effective strategies to mitigate PSMU and reduce its negative consequences.

Recently, NGOs and tech companies have shown interest in addressing problematic online use, including PSMU, by incorporating approaches that emphasize personal responsibility, usage moderation, self‐regulation, and increasing awareness.^10^ For instance, Google has launched a digital wellbeing (DWB) app to help users monitor and regulate their usage,^11^ and the Centre for Humane Technology (https://www.humanetech.com) has provided guidelines for the public, such as deleting toxic apps, fully disconnecting 1 day a week, and turning off notifications. Moreover, other approaches, such as software‐mediated tools, have been developed to increase user awareness of their digital habits and their impact.^12^, ^13^ However, there is a lack of research on the effectiveness of such apps and the perception and interests of users in using them.^14^ Moreover, their approaches focus on user‐centered solutions and neglect the role of technology design, which is loaded with persuasive techniques that effectively keep users hooked online.^15^

Social media design, which is characterized as interactive, personalized, and real‐time, employs persuasive principles that attract users’ attention and increase their engagement but also risk developing problematic behaviors.^16^, ^17^ In such instances, users may be unaware that they are being persuaded, which could impede their ability to recognize the persuasion attempts and control their actions.^18^ For example, features such as “likes” (social proof principle) and notifications of limited‐time posts (scarcity principle) can trigger a fear of missing out (FoMO) and encourage habitual platform use. FoMO can enhance preoccupation with social media,^19^ which, in turn, increases the difficulty in resisting excessive use, thereby increasing the risk of developing PSMU.^20^ Moreover, the constant accessibility of such platforms further adds to the challenge and makes it difficult for users to have self‐control regardless of their individual characteristics.^21^ In this context, there is a need to explore potential approaches beyond self‐control to mitigate PSMU.

One of the promising approaches for building resistance to persuasion is called attitudinal inoculation. This approach follows similar principles to medical inoculation.^22^ In order to strengthen people's immunity to persuasive attempts targeting their beliefs or attitudes, the theory advocates exposing them to a weakened version of persuasion attempts in the form of arguments (inoculation messages). In the context of PSMU, attitudinal inoculation could involve exposing adolescents to mild social media stimuli or arguments advocating for problematic use (PSMU symptoms in the current study), integrated with persuasion technique. These arguments are then accompanied by counterarguments promoting balanced usage habits. The idea is that by pre‐emptively challenging these arguments, individuals become aware and develop cognitive defenses that make them more resistant to PSMU development.

In this article, we propose the use of an inoculation‐based intervention in building resistance against persuasion found on social media and combat PSMU. Inoculation theory has traditionally been used to build resistance to attitude change, but more recent applications have expanded to influencing behavioral intentions and actions, particularly in health communication and misinformation domains. Our intervention highlights the symptoms of PSMU and the Cialdini's principles of persuasion commonly used on social media to create a sense of perceived threat. It then provides participants with strategies to resist these influences. The intervention aims to strengthen participants’ attitude toward reducing PSMU and potentially foster psychological resistance not just to persuasion but also to behavioral tendencies contributing to PSMU. This study represents a preliminary investigation designed to explore feasibility and identify potential trends to inform future, more comprehensive research. The inoculation theory has been applied successfully in various domains, including resistance to advertising,^23^ pressure to smoking,^24^ reactance to health messages,^25^ strategic pre‐crisis communication to foster confidence in government agencies,^26^ and misinformation on social media.^27^, ^28^ However, it has not been investigated yet within the domain of social media overuse, given that the nature of social media applications is persuasive and makes it easier to attract attention, spend more time on it, and potentially develop problematic behaviors.^16^ Additionally, the content of inoculation messages varies in literature. In our study, we explain both the persuasion techniques and the symptoms of problematic behavior. We posit that the application of an inoculation‐based intervention may offer a promising and effective strategy to mitigate PSMU.

### PSMU interventions

Different types of interventions and treatments emerged to combat generalized problematic internet use (PIU), which refers to the general excessive use of the internet, or specific forms of PIU, which refer to overuse or attachment to specific activities such as gaming or social media use (identified as PSMU in our study).^29^ For a detailed overview of such interventions, see the study in Ref. 30. These interventions were developed to focus on different aspects of problematic behavior of internet use, including interpersonal and health issues, time management, and compulsive and impulsive internet use.^31^, ^32^

### Technological interventions

Technological interventions primarily focus on regulating online usage. DWB apps, such as Google DWB,^11^ have been developed to help users balance their digital usage. These apps typically provide users with information about their usage amount, raise awareness of the negative impact of overuse, or offer self‐regulation features such as limit settings and notifications.^30^, ^33^ Other technical interventions, including browser extensions and nudges, have been designed to assist users. For example, a nudge was designed to minimize social media usage by utilizing techniques such as hiding notifications, limiting personalized content, and providing feedback on time usage.^34^ Moreover, through co‐design sessions, some studies have suggested modifications to existing digital platforms. These suggestions include introducing self‐control tools suitable for a multidevice ecosystem^35^ and making changes to the YouTube interface for a higher sense of agency.^36^ Nevertheless, there is a lack of research on the effectiveness of such tools and features, as well as limited insight into users’ perceptions and interests in using them.^14^ Furthermore, it is noteworthy that the majority of these interventions are directed toward users, placing less responsibility on digital designs or the designers.^37^

### Psychological interventions

As detailed in Refs. 30 and 38, different psychological treatments for generalized PIU, as well as PSMU, have been utilized with a primary emphasis on cognitive behavioral therapy (CBT). CBT, a technique widely used across diverse psychological conditions, has demonstrated effectiveness in treating both generalized PIU and specific PIU, such as gaming and social media.^39^, ^40^ CBT primarily operates by modifying behaviors through addressing and altering the underlying thoughts and beliefs. During CBT sessions, patients are taught to recognize maladaptive cognitions that trigger addictive behaviors while acquiring new coping strategies to prevent relapse. A typical CBT treatment course takes approximately 3 months and consists of two stages.^41^

Although CBT is the most supported psychological treatment in the literature, a variety of other psychological treatments, such as motivational interviewing, reality therapy, and positive technology, have demonstrated effectiveness in treating both generalized and specific PIU.^38^ However, psychological treatments for PIU are often expensive, require qualified counselors, and are typically employed after the problematic behavior has reached a risky level.^42^ It is still essential to investigate and develop alternative, more accessible, cost‐effective, and evidence‐based preventative and treatment strategies.

### Digital detox interventions

Digital detox refers to a period of time during which individuals disconnect from digital media or the online world and make efforts to restrict the use of digital tools.^43^ For example, the Smith & Jones Centre in Amsterdam, the first detox clinic for game addiction in Europe, has successfully employed traditional abstinence‐based treatment models.^44^ In China and South Korea, governments have established boot camps for internet addiction treatment,^45^, ^46^ incorporating physical training and military‐style obstacle courses. The effectiveness of digital detox interventions has yielded mixed findings.^47^, ^48^, ^49^ Additionally, although these interventions might be helpful in severe cases, they are not always practical solutions.^50^

### Pharmacotherapy interventions

Pharmacotherapy interventions involve exploring the potential benefits of medications that are typically used to treat other conditions for managing symptoms or underlying causes of PIU (both the generalized and specific forms).^51^ For instance, due to the common occurrence of comorbid depression among individuals with PIU, certain antidepressant drugs, such as fluoxetine and escitalopram, have been considered. These medications may help alleviate depressive symptoms and reduce the compulsive urge for repetitive internet use, consequently mitigating the strong urge to engage with the internet excessively.

It is important to highlight that pharmacotherapy is particularly aimed at individuals with PIU who have comorbid conditions, such as attention‐deficit/hyperactivity disorder and depression, and is more commonly utilized for issues related to problematic gaming rather than for the problematic use of social media.^52^ Furthermore, although some studies suggest the potential of this intervention in reducing online time and alleviating symptoms of PIU, it is important to acknowledge the limited scope of research in this area and highlight the preliminary nature of these findings.^53^

### Inoculation against problematic social media behavior

Attitudinal inoculation follows similar principles to medical inoculation.^22^ In order to strengthen people's resistance to persuasion, the theory advocates exposing them to a weakened version of the persuasion attempts in the form of inoculation messages. These messages must be balanced in a way that they are strong enough to evoke concern and a perceived threat that motivates resistance, but not so strong that they are overwhelming.^54^ Much like a flu vaccine, which introduces a weakened form of a virus to trigger the body's immune response, inoculation theory presents individuals with mild or weakened persuasive challenges, thus activating psychological resistance. This exposure stimulates protective responses motivated by a perceived threat that help individuals defend their existing beliefs or attitudes against subsequent persuasive challenges or attacks. Inoculation theory was initially developed to protect one's beliefs and attitudes against persuasive influence,^22^, ^54^, ^55^ and recently, it has been extended to influence behavioral intentions and actual behaviors, especially in health and public communication domains.^25^, ^56^, ^57^ Traditionally, this approach (known as prophylactic inoculation [preventive]) has focused on reinforcing existing positive attitudes. A key feature of prophylactic inoculation is its alignment with recipient's initial positive attitudes—the message affirms the existing belief while defending it against foreseeable counterarguments.^58^ Later scholars have extended the framework to include therapeutic inoculation—used not to prevent but to treat, which targets individuals with neutral or opposing attitudes and aims to shift their views and protect those newly formed attitudes against future persuasion.^58^, ^59^, ^60^, ^61^ Building on these theoretical foundations, researchers have applied inoculation across various contexts to evaluate its real‐world effectiveness. Inoculation has been utilized in different domains; for example, a study by Roozenbeek and van der Linden^62^ demonstrated the effectiveness of attitudinal inoculation against online misinformation by exposing participants to the role of a fake news creator and learning about six documented techniques commonly used in misinformation production. Another recent study applied inoculation theory in the online gambling domain to build resistance against persuasive interfaces. These findings demonstrate how the use of explainable persuasion through the disclosure of persuasive techniques can inoculate individuals against the influence of such interfaces.^63^

Attitudinal inoculation posits two key components that contribute to building resistance: threat and refutational pre‐emption.^54^ The threat component functions as the motivational catalyst; it is the recognized vulnerability of a position that motivates defensive responses.^64^ This threat can be triggered through explicit forewarnings, which directly alert individuals to the likelihood that their current attitudes may be challenged or attacked, or through exposure to counterarguments or opposing views (implicit threat).^54^, ^60^, ^65^ Although forewarnings are frequently included in inoculation messages and some scholars have previously treated forewarning and threat as interchangeable, they are not required in inoculation messages.^55^ Threat is better understood as a response to perceived vulnerability, not as a feature of the message itself. Compton and Ivanov^64^ argue that forewarnings are primarily responsible for generating threat. Nonetheless, messages that incorporate both explicit forewarnings and implicit threat cues tend to generate better inoculation effects.^66^ Building on this understanding, studies have explored the underlying mechanisms through which threat contributes to inoculation‐induced resistance to persuasion.^67^ Researchers have examined whether threat primarily operates through generating apprehension (e.g., anxious or fearful) or through creating a motivational state that encourages defending attitudes. Findings suggest that conceptualizing threat as motivation, rather than mere apprehension, aligns more with the threat function in conventional inoculation theory.^67^ Additionally, studies have demonstrated that both apprehensive and motivational threats can be elicited by inoculation messages and may persist after exposure to persuasive attacks.^68^, ^69^

Although threat is important in initiating resistance, research suggests that the combined presence of both threat and refutational pre‐emption is more effective in promoting inoculation effects against persuasion.^70^ The refutational pre‐emption component involves providing individuals with ways or information to refute such persuasive attempts, which can help protect and strengthen their attitude against future persuasive challenges.^71^ In other words, the threat, through exposing individuals to the opposing argument or viewpoint, is perceived as an incoming attack or persuasion attempt that challenges one's existing attitude. The threat serves as a motivational force to protect attitude against attacks and confer resistance. Motivated by the perceived threat, individuals can use the counterarguments provided by refutational pre‐emption to refute future persuasion attempts.^56^ Refutational pre‐emption also offers individuals a way to practice defending their attitude using the provided counterarguments. Refutational pre‐emptions may also vary in structure. Refutational‐same treatments are tailored to address persuasive challenges the individual might later encounter, whereas refutational‐different treatments address alternative arguments that are not included in the subsequent persuasive challenges. Both formats have shown to be effective in promoting resistance,^54^, ^70^ yet findings remain mixed. Some studies indicate stronger resistance when the same arguments are later encountered,^22^ whereas others report either no significant difference^72^ or better results with refutational‐different formats.^73^ These findings suggest that inoculation messages can still confer resistance, even when future persuasive challenges are not anticipated.

Inoculation messages can trigger attitudinal resistance through either cognitive or affective approaches. Although early inoculation theory emphasized cognitive mechanisms as central to building resistance, later studies have highlighted the role of affective strategies that generate resistance through emotional responses. For example, a study comparing cognitive‐based and affective‐based inoculation messages found no significant difference in their resistance outcomes, suggesting that both approaches can be effective in building resistance against persuasion.^64,74^ Research also indicates that the effectiveness of inoculation messages increases when the message type aligns the individual's attitude base—cognitive messages are more effective for cognitively driven attitudes, whereas affective messages work better when attitudes are emotionally based.^75^ Furthermore, the effectiveness of inoculation interventions has been evaluated across different mediums, including printouts,^76^ videos,^77^ and game‐based strategies.^62^, ^78^ A comparative study investigating the medium used for delivering the inoculation message revealed that print and video modalities did not exhibit significant differences in fostering resistance, although they operate through different mechanisms.^79^

As attitudinal inoculation has been explored in the literature, two primary approaches have been identified for refutational pre‐emption: passive and active.^22^ The passive refutations (passive inoculation) are operationalized by exposing participants to refutational content provided by researchers. On the other hand, active refutations (active inoculation) are operationalized by requiring participants to generate their own counterarguments to the persuasive attempts presented in the inoculation message. Studies have shown different results on which method is more effective than the other. Some studies showed that passive inoculation was superior in conferring resistance compared to active inoculation.^80^ A potential explanation for these findings is the less cognitive demand required during the process of this method and the potentially better quality of counterarguments.^70^ Other studies have shown that active inoculation was superior at conferring resistance compared to passive inoculation.^81^ These findings were explained by the cognitive and engagement process required during the active inoculation, which helped generalize resistance to persuasion.^82^ Furthermore, some studies suggested that the effectiveness of the inoculation process may be influenced by issue involvement.^56^ Issue involvement is a measure that evaluates the relevance or importance of an attitude object to an individual.^83^ For effective inoculation intervention, it has been argued that optimal levels of issue involvement are necessary, as the extent of issue involvement can influence an individual's response to threat messages.^55^, ^84^ Those with high issue involvement are often less likely to be influenced by the threat messages due to their keen interest in the issue. Conversely, individuals with low issue involvement may perceive the topic as irrelevant and lack the motivation to engage in the defense‐building process. In our study, we examine both passive and active inoculation strategies, while also measuring issue involvement to account for its potential effect.

Previous studies have identified the impact of inoculation intervention on key factors, including resistant attitudes and post‐inoculation talk (PIT) (also referred to as word‐of‐mouth communication).^85^, ^86^ Compton and Pfau^87^ advanced a theoretical case for PIT suggesting that inoculation interventions operate at an interpersonal level and motivate inoculated individuals to talk about the inoculation message with others. PIT occurs when individuals discuss the persuasive attempt or related topic with others, potentially spreading the inoculation effect beyond those involved in the inoculation process.^88^ Although traditional inoculation theory emphasized subvocal counterarguing, which is an internal process of coming up with arguments to defend against future persuasion, PIT has been conceptualized as vocal counterarguing that plays a role in the inoculation process. Therefore, individuals not only defend their beliefs or attitudes mentally but also engage in conversations with others that either reassure their current attitude triggered by perceived threat or advocate their positions, especially by using the content provided through refutational pre‐emption, thereby strengthening attitudinal resistance through social reinforcement.^87^ Supporting this theoretical implication, Ivanov et al.^89^ showed that recipients of an inoculation message talked about the topic compared to the control group and that the PIT statistically mediated greater resistance to a later persuasive attack. Clear et al.^90^ also found that individuals exposed to inoculation were more inclined to discuss the inoculation message with others post‐inoculation intervention. Further studies analyzed PIT and found that inoculated individuals not only recirculated the inoculation content but also generated new supportive arguments and engaged in advocacy, indicating that PIT can both reassure speaker's own attitude and propagate resistance outward.^85^, ^91^

These findings underscore the potential role of PIT not only as an outcome of inoculation but also as a mechanism that actively strengthens resistant attitudes.^89^ For instance, Compton and Pfau^86^ found that inoculation intervention increased resistance to credit card marketing by protecting existing positive attitudes about credit card debt, promoting healthy credit card behavior, and impacting word‐of‐mouth communication about credit card debt. Accordingly, it is important to investigate whether inoculation intervention, in addition to its potential effect on the attitude toward PSMU, increases the likelihood of engaging in PIT as part of the post‐inoculation intervention behavior.

Recent developments in inoculation theory have introduced the use of booster messages, which are secondary exposures delivered after the initial inoculation as a strategy to strengthen and prolong resistance to persuasion. Analogous to medical booster doses, message boosters are designed to further enhance one's ability to defend their attitude. It is important to note that booster messages were not part of McGuire's^22^, ^54^ original conceptualization of inoculation theory. Rather, they represent a contemporary extension of the framework, supported by growing empirical evidence. Although some literature offers mixed results regarding their effectiveness,^23^ Pfau et al. suggested that the timing of boosters is important for their effectiveness.^92^ One of their studies revealed that a booster session delivered 70 days after the initial inoculation had a minimal effect.^24^ Conversely, a booster delivered between 1 and 3 weeks after the initial inoculation was found to sustain the effectiveness of the inoculation for an extended period of more than 6 weeks.^92^ Compton and Pfau,^56^ however, suggested exploring different forms or designs of booster sessions. Supporting this idea, a study conducted by Ivanov et al.^93^ showed the potential of using a booster session in the form of a second inoculation message. Drawing from these studies, the present study incorporated booster sessions as a second session following the initial inoculation, aiming to reinforce the primary message.

Based on the preceding background study, this study aims to address the following research questions:

RQ1: Can inoculation intervention (passive and active types) confer resistance against PSMU?

RQ2: Can inoculation intervention (passive and active types) affect (a) attitude toward PSMU, (b) likelihood to engage in positive PIT about PSMU, and (c) likelihood to engage in negative PIT about PSMU?

## METHODS

### Study design

The present study employed a 3 × 2 mixed design to evaluate the effect of an inoculation intervention in conferring resistance to PSMU. The inoculation intervention was delivered through a set of scenarios presented in a booklet, encompassing both textual and image descriptions. The intervention groups, comprising three experiential conditions (passive inoculation, active inoculation, and control), correspond to the between‐subjects factor. Time measurements were collected (pre‐intervention and post‐intervention) as part of the within‐subjects factor. PSMU was measured as the dependent variable, with baseline issue involvement serving as a covariate. Additionally, attitude toward PSMU and PIT were also analyzed separately as dependent variables. The inoculation scenarios and dataset used for this study are available at the Open Science Framework (see Data Availability Statement).

### Participants

A total of 50 school students from an international school in Qatar participated in the study, which was conducted in‐person within the school premises. The students had access to laptops to complete the online survey part of the study. The inoculation materials, scales used to measure variables, and the overall study design were in English, aligning with the international school's use of English as the primary medium of education.

The inclusion criteria for participants required individuals to be between the ages of 11 and 15 and to be users of social media. The participants were grouped into

- Passive inoculation group (threat with passive refutations): Students received both components of the inoculation intervention message: the threat and the refutational pre‐emption.
- Active inoculation group (threat with active refutations): Students were provided with threat messages and then asked to list their strategies for addressing each scenario, thereby generating their own refutational pre‐emptions.
- Control group: Students were not given any specific activity between the pre‐intervention and post‐intervention measurements.

### Measures

#### PSMU

PSMU was measured using social media disorder (SMD) scale.^94^ The scale comprises nine items developed using the diagnostic criteria for internet gaming disorder (GD) defined in DSM‐V. The nine items of the scale measure nine symptoms of SMD behaviors, including preoccupation, persistence, tolerance, withdrawal, displacement, escape, problems, deception, and conflict. Each item was rated on a 5‐point Likert scale (1 = never to 5 = always). The scale was used in literature with school‐age groups, for instance, on US participants aged 13–19 years^95^ and on Turkish participants aged 14–18 years.^96^ The scale demonstrated good internal consistency. Cronbach's alpha in the current study was 0.79 for each pre‐intervention and post‐intervention, indicating good reliability.

#### Issue involvement

Issue involvement was assessed using an abbreviated version of the Personal Involvement Inventory developed by Zaichkowsky,^83^ a scale that is widely employed in inoculation research.^55^, ^63^, ^84^, ^86^ The scale comprises six items characterized by bipolar adjectives: unimportant–important, irrelevant–relevant, of no concern–of concern to me, means nothing–means a lot, does not matter–matters to me, and insignificant–significant. Participants were asked to rate their sense of importance on the topic of being too attached to social media using a 7‐point Likert scale. The reliability ratings for issue involvement in the current study demonstrated high internal reliability with Cronbach's alpha values of 0.95 for pre‐intervention and 0.97 for post‐intervention.

#### Attitude

Participants’ attitudes toward problematic attachment to social media were assessed during pre‐intervention and post‐intervention using an attitude scale developed by Burgoon et al.^97^ The scale comprises six bipolar adjective pairs on a 7‐point scale: wrong–right, negative–positive, bad–good, unfavorable–favorable, unacceptable–acceptable, and foolish–wise. The reliability ratings for attitude in current study demonstrated high internal reliability with Cronbach's alpha values of 0.94 for pre‐intervention and 0.87 for post‐intervention.

#### Post‐inoculation talk (PIT)

PIT was assessed using two items, one for positive word‐of‐mouth (referred to as positive PIT in this study) and another for negative word‐of‐mouth (negative PIT), adapted from “the effects of party‐ and pac.”^98^ Participants were asked to rate the likelihood that they would discuss the topic either positively or negatively on a scale ranging from 0 (no probability) to 100 (certain probability).

### Pilot test

A pilot test was conducted face‐to‐face before conducting the main experiment and involved two adolescents. The pilot test was also important to validate the scales to ensure cultural fitness and clarity of questions. The outcomes of the pilot test led to several modifications to enhance the survey questionnaires and scenarios. Specifically, adjustments were made to the language, grammar, and sequencing of sections to ensure that participants could easily comprehend the scenarios.

### Procedure

**FIGURE 1:**
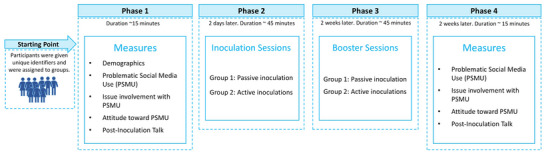
Inoculation study procedure.

Prior to conducting the study experiment, meetings were conducted with the school administration to explain our research objectives and to coordinate the logistical aspects of conducting the study. In alignment with ethical research practices, informed assent was obtained from the participating students, alongside consent from their parents or legal guardians. The Institutional Review Board approval was obtained from Hamad Bin Khalifa University (QBRI‐IRB 2021‐08‐104). Additionally, we verified that none of the groups received special training related to PSMU during the study period. The study consisted of four phases, as shown in Figure 1.

#### Phase 1

In the first phase, the participants responded to a questionnaire that assessed their level of PSMU, issue involvement, attitude toward PSMU, and PIT (likelihood to engage in PSMU word‐of‐mouth [positive and negative]). The participants were also asked to provide their background information.

#### Phase 2

Phase 2 was conducted 2 days after Phase 1, a strategic decision aimed at minimizing potential for a priming effect and reducing the risk of participant fatigue that could result from conducting the survey immediately prior to the inoculation sessions. The priming effect is a phenomenon wherein previous encounters with certain stimuli influence reactions to subsequent stimuli based on the principle of memory accessibility.^99^ The priming effect posits that initial stimuli enhance the recallability of related information when encountering later stimuli. In the context of our study, such priming could inadvertently occur if the survey questionnaire, which was related to PSMU, potentially influenced participants’ reactions and responses to the inoculation session. In Phase 2, the inoculation intervention was delivered in the school's classrooms. The participants were randomly assigned to either the passive inoculation or the active inoculation group. Each group was facilitated by two instructors, one of whom was the first author who delivered the inoculation intervention in a session that lasted about 45 min for each group.

Before commencing the session, the participants were briefed on the session's flow and were reminded to provide their responses freely because data were collected anonymously. Instructors walked through each scenario with the participants to elicit threats about PSMU. After each scenario, participants were given time to answer the follow‐up questions. Subsequently, in the passive inoculation group, participants were guided through the “refutational pre‐emption,” the set of methods to refute each scenario. In the active inoculation group, the participants were asked to list ways they could deal with each scenario.

#### Phase 3

Phase 3, referred to as a booster session, occurred 2 weeks after the initial inoculation session in Phase 2. Participants were presented with scenarios that were similar, but not identical, to those introduced in Phase 2 (see Supporting Information). The session was conducted in a similar setting and followed a similar flow to Phase 2.

#### Phase 4

Phase 4 occurred 2 weeks after Phase 3. The literature suggested a need for a delay to measure effectiveness after the inoculation intervention.^70^ In Phase 4, participants responded to the same set of questionnaires as in Phase 1, which assessed their level of PSMU, issue involvement, attitude toward PSMU, and PIT (likelihood to engage in PSMU word‐of‐mouth [positive and negative]).

##### Materials

**FIGURE 2:**
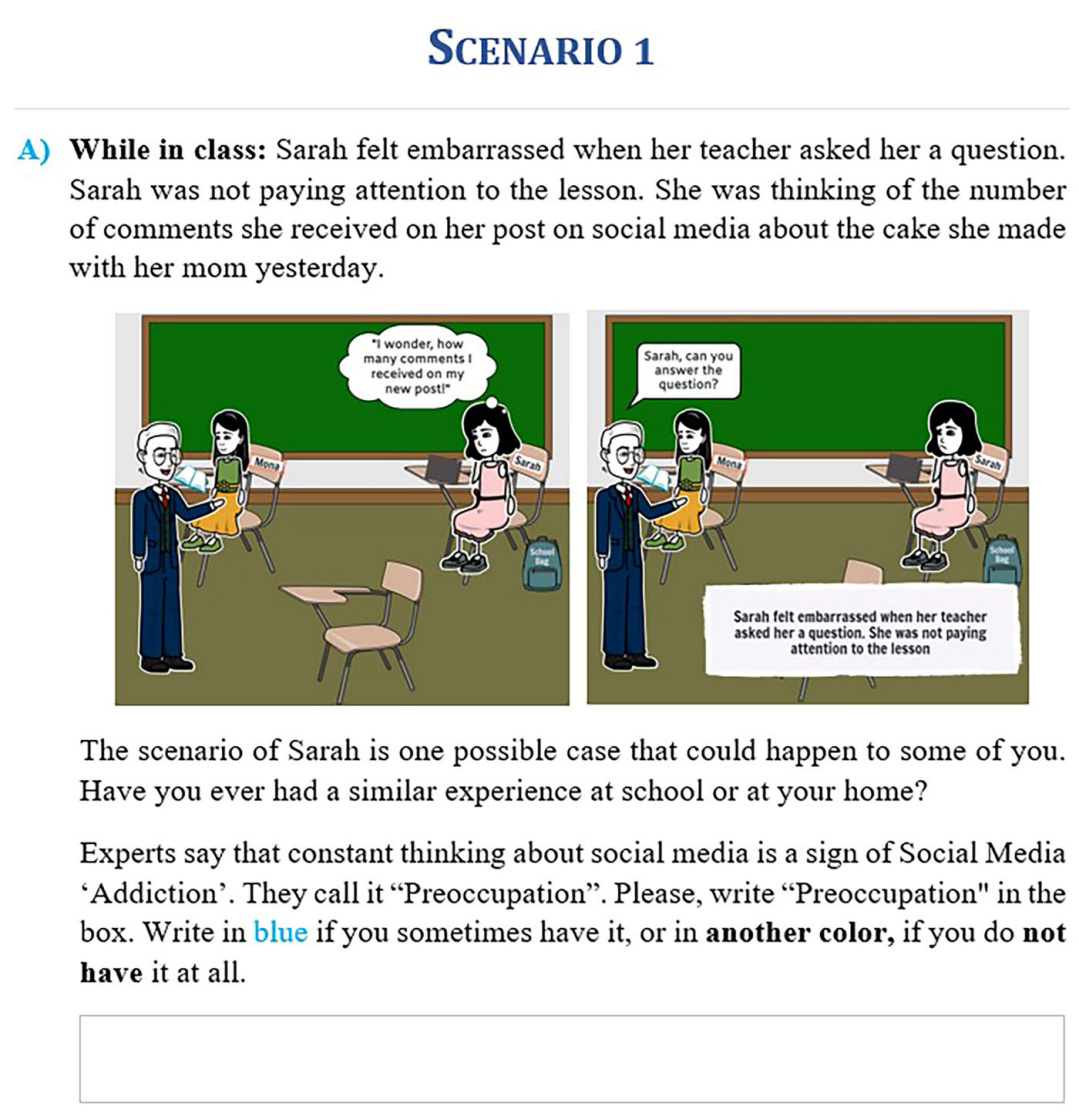
Example of the inoculation intervention scenarios.

The inoculation intervention script comprised six inoculation scenarios designed to elicit threats of potential manipulation of social media overuse on one's behavior. The inoculation scenario consisted of two main components. The first primary component was designed in the form of scenarios with messages representing weak versions of the problematic use of social media, characterized by the nine symptoms of SMD: preoccupation, tolerance, withdrawal, persistence, displacement, problem, deception, escape, and conflict. To ensure participant engagement and attention, they were prompted to annotate each identified symptom in the scenario in a specific color if they had experienced it and in another color if they had not, thus enhancing their interactive involvement with the material. Previous studies have shown that information visualization that uses both verbal and nonverbal codes can provide recipients with a better opportunity to remember and process the information compared to using only one of these two codes.^100^ This is because the human brain operates with two different types of mental representation: verbal and nonverbal codes. Therefore, the scenarios were designed in the form of text and images, as shown in Figure 2.

Inoculation scenarios aimed to trigger participants’ cognitive processes and make them aware of their thoughts and attitudes toward social media overuse. The participants were then asked follow‐up questions to rate each scenario for how they relate to it, their level of knowledge on how to avoid it, and finally, their level of confidence in responding to it. For instance, following Sarah's scenario depicted in Figure 2, follow‐up questions included “How much does Sarah's scenario relate to you?” with responses measured on a 5‐point Likert scale (1: Does Not Relate at All, 5: Extremely Related), “Do you know how to avoid Sarah's situation happening to you?” with “Yes/No” responses, and “How confident are you in dealing with Sarah's situation if it happens to you?” with responses measured on a 5‐point Likert scale (1: Not at All Confident, 5: Extremely Confident). Participants were also prompted with an open‐ended question for listing their thoughts after each scenario. These questions shall further stimulate a higher level of cognitive process and elaboration of the inoculation scenarios. This is supported by a previous study that analyzed variables influencing high‐thinking processes, including self‐relevance, knowledge, and confidence.^101^ In addition, it has been demonstrated that asking participants to write comments after the inoculation messages enhanced the effect of the inoculation.^102^

**FIGURE 3:**
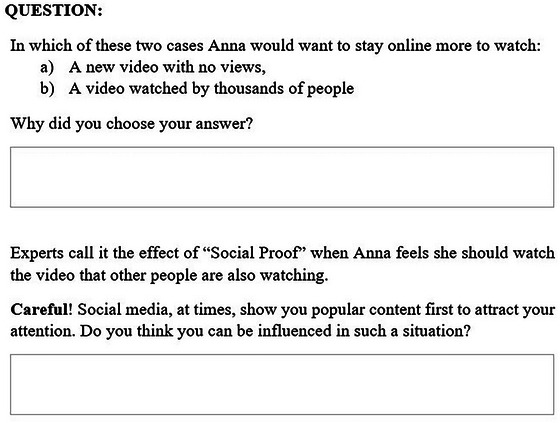
Example of the Cialdini persuasive principle in the inoculation message.

Each scenario has also reflected a persuasive technique adapted from the Cialdini principles of persuasion,^103^ as shown in Figure 3. Cialdini principles, which are broadly used persuasive techniques, are found in digital media designs, including social media.^17^, ^104^ The principles include reciprocity (people tend to pay back favors done to them), liking (people are persuaded more by what they find attractive or familiar), social proof (people do what they observe other people doing), scarcity (people are more likely to be influenced by things that are limited or available for a short time), authority (people are more likely to comply with figures of authority, experts, or celebrities), and commitment and consistency (people are more likely to be consistent in their behavior and actions; people's tend to be consistent with their first opinion). Through the lens of these principles, the design features of social media exploit psychological susceptibilities, leading to immersive and prolonged engagement with these platforms. For example, notifications about limited‐time posts, trending topics, and active conversations can trigger FoMO, which, in turn, leads to constant engagement with the platforms.^105^ This heightened state of FoMO can develop a preoccupation with social media, a symptom of PSMU.^19^ The “like” feature, as explored by Sherman et al.,^106^ serves as a form of social proof. The study found adolescents favor posts with more “likes” even those endorsing risky behaviors, enhancing social media's persuasive effect by reinforcing the desire for social belonging. Such mechanisms increase user engagement that potentially develops the risk of PSMU by encouraging a continuous search for approval and FoMO on social interactions.

**FIGURE 4:**
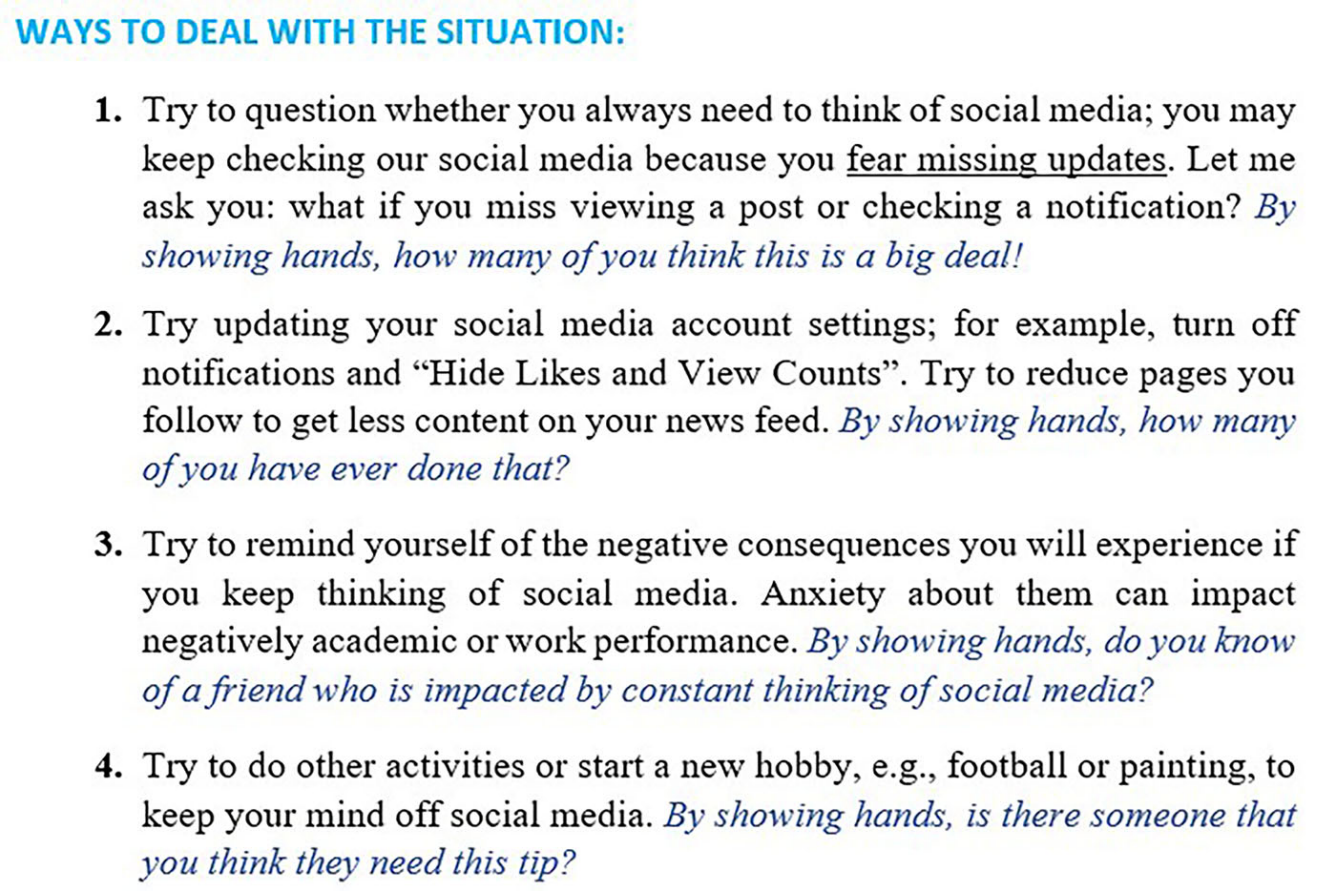
Example of the refutational pre‐emption of the passive inoculation.

The second primary component of the inoculation intervention was the inclusion of refutational pre‐emption. The refutational pre‐emption provides participants (passive inoculation group) with counterarguments to refute the symptoms of PSMU and potentially combat PSMU. For each scenario, a set of strategies or information was presented to address the PSMU, the symptom raised in that particular scenario. These counterarguments were formulated based on existing literature and advice from online psychologists. Furthermore, they underwent a revision process facilitated by a psychologist. An example of the inoculation refutational pre‐emption is shown in Figure 4.

### Data analysis

In preparing the data for analysis, participants whose time spent online was controlled by their parents were not included in the study in order to ensure that our findings reflect the effects of the attitudinal inoculation intervention on individuals making autonomous decisions regarding their online behavior—thereby avoiding any bias introduced by external control. Data normality was checked using skewness and kurtosis values. ^107^ The scales assessing PSMU, attitude toward PSMU, and PIT exhibited skewness and kurtosis between ±2, indicating an approximately normal distribution. Wilcoxon signed‐rank tests (two‐tailed) were used to compare PSMU symptoms across the intervention groups. Mixed ANCOVA analyses were performed to test the effect of the inoculation intervention across the three groups, with issue involvement as a covariate. All statistical tests were conducted using a significance threshold of *p* < 0.05. Data were analyzed using JASP version 0.17.^108^

## RESULTS

### Descriptive statistics

Descriptive statistics of the intervention groups are summarized in Tables 1 and 2 . The study included a total of 50 participants divided into three groups: passive (*N* = 20), active (*N* = 20), and control (*N* = 10). The mean age of participants was 13.25 (SD = 1.33) for the passive group, 12.95 (SD = 1.32) for the active group, and 14.50 (SD = 0.71) for the control group. In terms of gender distribution, the passive group consisted of 40% males and 60% females, the active group consisted of 60% males and 40% females, and the control group consisted of 30% males and 70% females.

**TABLE 1:** Participant demographics.

Variables		Passive *N* (20)	Active *N* (20)	Control *N* (10)
Age	Mean (SD)	13.25 (1.33)	12.95 (1.32)	14.50 (0.71)
Gender	Male Female	8 (40.00%) 12 (60.00%)	12 (60.00%) 8 (40.00%)	3 (30.00%) 7 (70.00%)

**TABLE 2:** Descriptive statistics of PSMU, attitude toward PSMU, PIT, and issue involvement.

Variables		Passive	Active	Control
		Mean (SD)		
PSMU	Pre Post	20.20 (5.44) 18.00 (5.20)	21.30 (4.43) 17.85 (3.95)	18.40 (7.62) 18.20 (6.96)
Attitude toward PSMU	Pre Post	2.98 (1.18) 2.64 (1.03)	2.35 (1.06) 2.68 (0.89)	3.00 (1.14) 2.53 (1.03)
PIT positive	Pre Post	40.00 (23.40) 35.00 (22.12)	32.50 (21.98) 22.50 (19.70)	38.00 (30.48) 28.00 (27.41)
PIT negative	Pre Post	51.00 (24.69) 63.50 (23.23)	52.00 (23.75) 54.00 (22.10)	49.00 (23.31) 42.00 (26.58)
Issue involvement	Pre (baseline)	4.57 (1.31)	3.92 (1.31)	4.10 (1.10)

Abbreviations: PIT, post‐inoculation talk; Post, post‐inoculation intervention; Pre, pre‐inoculation intervention; PSMU, problematic social media use.

**TABLE 3:** Wilcoxon signed‐rank test for problematic social media use (PSMU) symptoms within each group.

	Passive			Active			Control		
Variable	*Z*	*p*	Rank‐biserial correlation	*Z*	*p*	Rank‐biserial correlation	*Z*	*p*	Rank‐biserial correlation
Withdrawal	1.27	0.183	0.46	1.96	0**.037**	0.67	−0.14	0.942	−0.06
Persistence	1.26	0.222	0.50	1.97	0**.045**	0.56	0.93	0.386	0.39
Escape	0.21	0.851	0.07	0.71	0.478	0.24	−1.60	0.174	−1.00
Displacement	0.94	0.343	0.31	2.67	0**.003**	1.00	0.54	0.773	0.33
Problem	0.98	0.336	0.33	1.26	0.197	0.40	−0.59	0.588	−0.25
Preoccupation	0.34	0.777	0.14	1.51	0.128	0.46	−1.60	0.149	−1.00
Tolerance	1.50	0.115	0.47	−0.27	0.813	−0.09	0.73	0.484	0.33
Deception	2.19	**0.025**	0.82	2.49	0**.012**	0.85	0.37	0.850	0.20
Conflict	0.80	0.393	0.27	1.54	0.096	0.47	1.28	0.265	0.70

*Note*: Bold values indicate statistically significant results at p < 0.05.

### Problematic social media use and inoculation

A 3 × 2 mixed ANOVA was conducted to examine the effect of the inoculation intervention on PSMU, measured two times: pre‐intervention and post‐intervention. The baseline issue involvement was controlled. Within‐subjects analysis for the main effect of time point was not statistically significant (*F*(1,46) = 1.92, *p* = 0.173, partial *η* ^2^ = 0.04). However, the interaction effect between the time point and intervention group was significant (*F*(2,46) = 3.37, *p* = 0.043, partial *η* ^2^ = 0.13), indicating a large effect size. Between‐subjects analysis revealed no significant main effect of group on PSMU (*F*(2,46) = 0.33, *p* = 0.722, partial *η* ^2^ = 0.01), indicating a small effect size. Additionally, pre‐issue involvement significantly influenced PSMU (*F*(1,46) = 6.69, *p* = 0.013, partial *η* ^2^ = 0.13), indicating a large effect size, with a non‐significant interaction between pre‐issue involvement and group (*F*(1,46) = 1.89, *p* = 0.176, partial *η* ^2^ = 0.04).

A follow‐up post hoc analysis indicated that the PSMU score before the active inoculation intervention (*M* = 21.30, SD = 4.43) was significantly higher than after the intervention (*M* = 17.85, SD = 3.95). No significant differences were observed in PSMU scores (pre‐intervention and post‐intervention) for the other groups.

Further analysis was conducted to examine the effect of the intervention on each symptom of PSMU within each study group, as presented in Table 3. In the passive group, significant effects were observed in the deception symptoms, suggesting that passive inoculation influenced users’ tendencies toward deceptive behaviors related to social media use. Conversely, the active group showed significant improvements in withdrawal, persistence, displacement, and deception, indicating an effect of the intervention on these aspects of PSMU. The control group did not exhibit significant changes across the symptoms.

### Attitude toward PSMU

A mixed ANOVA was performed to assess changes in the attitude toward PSMU across different time points (pre‐ and post‐intervention) and groups. The within‐subjects analysis indicated that the main effect of time point was not statistically significant (*F*(1,47) = 1.53, *p* = 0.223, partial *η* ^2^ = 0.03), indicating that the overall attitude toward PSMU did not significantly change over time for the entire sample. However, there was a significant interaction between the time point and group (*F*(2,47) = 4.25, *p* = 0.020, partial *η* ^2^ = 0.15). This interaction suggests that the effect of the intervention on attitude toward PSMU varied among the groups. Nonetheless, the between‐subjects analysis did not show a significant main effect of the group on the attitude (*F*(2,47) = 0.52, *p* = 0.599, partial *η* ^2^ = 0.02). Further, subsequent post hoc analyses did not yield significant results across all groups.

### PIT

A mixed ANOVA was conducted on both negative PIT and positive PIT separately in relation to PSMU post‐inoculation intervention. For the negative PIT, within‐subjects analysis revealed that the main effect of time point was not statistically significant (*F*(1,47) = 0.46, *p* = 0.502, partial *η* ^2^ = 0.01). Additionally, there was no significant interaction between the time point and group (*F*(2,47) = 2.22, *p* = 0.120, partial *η* ^2^ = 0.09). The between‐subjects analysis also indicated no significant main effect of the group on negative PIT (*F*(2,47) = 1.12, *p* = 0.334, partial *η* ^2^ = 0.05). Nonetheless, a subsequent simple main effect revealed that the negative PIT measured pre the passive inoculation intervention (*M* = 51.00, SD = 24.69) was marginally significantly lower than the negative PIT measured post the passive inoculation intervention (*M* = 63.50, SD = 23.23) (*F*(1) = 4.25, *p* = 0.053). There were no significant differences between the two measures of PSMU (pre‐intervention and post‐intervention) for the other groups.

In the analysis of positive PIT on PSMU, the within‐subjects analysis showed that the main effect of time point was statistically significant (*F*(1,47) = 6.20, *p* = 0.016, partial *η* ^2^ = 0.12). There was no significant interaction between the time point and group (*F*(2,47) = 0.30, *p* = 0.744, partial *η* ^2^ = 0.01). The between‐subjects results also revealed no significant main effect of the group on positive PIT (*F*(2,47) = 1.19, *p* = 0.313, partial *η* ^2^ = 0.05).

A subsequent analysis of the simple main effects demonstrated marginal significance in positive PIT before the active inoculation intervention (*M* = 32.50, SD = 21.98) in comparison to after the intervention (*M* = 22.50, SD = 19.70) (*F*(1) = 3.52, *p* = 0.076). There were no significant differences observed in the positive PIT measures (pre‐intervention and post‐intervention) for the remaining groups.

## DISCUSSION

The application of inoculation theory to combat attitudinal change or behavioral susceptibilities is not new. Historically, its roots can be traced back to persuasion literature where individuals were metaphorically “inoculated” against opposing arguments to make them more resilient against subsequent persuasion attempts.^22^ In the context of PSMU, this study explored a relatively unexamined area using this theoretical framework. The results shed light on the impact of such interventions on PSMU, attitudes toward PSMU, and PIT, with baseline issue involvement serving as a covariate.

In examining the effect of the inoculation intervention on PSMU, the findings revealed that the inoculation intervention effectively reduced the level of PSMU post‐intervention for the active inoculation group. In contrast, the passive inoculation and control groups did not show significant change. These findings demonstrate the potential efficacy of the inoculation intervention in active formats in influencing PSMU behaviors.

Nevertheless, in exploring such significant differences in the symptoms associated with PSMU, the active inoculation group outperformed its passive counterpart. The active group exhibited more significant changes in symptoms such as withdrawal, persistence, displacement, and deception. The passive inoculation group showed a significant difference in the deception symptom only. These findings suggest the increased efficacy of active inoculation, which requires participants to engage more deeply with the study material and foster a critical evaluation of their PSMU behaviors. The findings could be explained by the cognitive and engagement process involved during the active inoculation, which might facilitate a generalized or broader resistance.^82^ Our study was tested in a “refutational‐different” format, where participants were exposed to scenarios that were similar, but not identical, to other potential scenarios they might encounter post‐inoculation, which could enhance the generalizability of the inoculation's effectiveness.^109^ Such an approach likely helped them recognize PSMU and its symptoms on a more general level and actively resist various related scenarios. In addition, although the passive inoculation group did not show a significant reduction in the overall PSMU score (a reduction in the mean value was observed), the changes in specific symptoms were also less pronounced than in the active group.

In our study examining how inoculation intervention impacts attitude resistance and the likelihood of spreading PIT, we found that the results were inconclusive. In our findings, although there was no significant main effect of the inoculation intervention on participants’ attitudes toward PSMU, there was a significant interaction between time (pre‐ and post‐) and the intervention group. Nevertheless, there were no significant differences observed in the post hoc analysis. One possible explanation could be that participants’ initial attitude was already low, which might impact the potential to observe significant change or decrease in attitudes if participants had more positive attitudes to PSMU. This is in‐line with a previous study that showed that a high value for initial attitude could impact the change in the post‐intervention attitude.^110^

The results pertaining to PIT post‐inoculation were intriguing. Although there was no significant main effect or interaction effect for negative PIT, the passive inoculation group demonstrated a marginally significant increase in negative talk post‐intervention. Additionally, the active inoculation group showed a marginal decrease in positive talk post‐intervention. These findings resonate with previous research that has highlighted the role of inoculation in enhancing word‐of‐mouth communication.^89^ The previous research suggests inoculation motivates PIT among social networks, which, in turn, enhances a resistant attitude.^87^ In our findings, no significant difference was observed in either variable. Nevertheless, further research is needed to explore other impacting factors, such as source credibility.^111^ It is suggested that the source credibility and perceived trustworthiness of those delivering the inoculation can impact the PIT, which was not measured in the present study.

Overall, the findings from the present study demonstrated that an attitudinal inoculation‐based intervention may offer a potential cost‐effective method to combat PSMU. The intervention could also be explored for other problematic online behaviors, such as GD, as well as for different age groups. Although GD and PSMU share psychological features like compulsion and reward‐seeking behaviors, each presents its unique characteristics.^112^ For example, GD often involves discrete gaming sessions with clear start and end points, whereas social media use is a more continuous activity and demands emotional involvement.^113^ Future studies could consider adapting our inoculation intervention approach to GD, considering its distinct characteristics. Additionally, it is also important to note, as critiqued in recent literature, that the efficacy of such inoculation strategies may be contingent upon the individual's level of attitude certainty and confidence. Specifically, although these strategies can be effective for those with high attitude certainty and confidence, they might prove counterproductive for individuals with low levels of these attributes.^114^

Nevertheless, the findings should be interpreted within the context of its limitations. Among them, recall bias and the influence of social desirability were potential limitations. Efforts to mitigate these biases included implementing a waiting period of 2 days between Phases 1 and 2. Moreover, the interval of 2 weeks for the post‐intervention assessment, while limited, was deemed appropriate given the study's aim to assess potential rather than actual effects of the intervention. Additionally, the study was limited in statistical power due to a relatively small sample size. The sample, drawn from international schools, could potentially comprise individuals from more diverse educational and socio‐economic statuses. Participants reported a low level of baseline attitude toward PSMU, potentially being affected by social desirability bias. Such reported scores may have hindered the ability to observe attitude changes post‐inoculation intervention. Another limitation was the lack of an elicited threat measure. This study was exploratory, with a primary objective to evaluate participants’ PSMU behavior after being exposed to active and passive inoculation conditions, with less of a focus on understanding the underlying processes driving the resistance to persuasion. Consequently, any presence of a threat can only be inferred. However, this is a significant theoretical limitation as perceived threat is what differentiates inoculation from other approaches like literacy training. Recent work by Kuru^115^ provides evidence that forewarning, the motivational component of threat, is the defining feature that distinguishes inoculation from literacy‐based interventions and yields better resistance outcomes. Without a threat measure, we cannot confidently attribute observed effects to inoculation processes. Moreover, the type of threat also matters. As Banas and Richards^67^ argue, motivational threat (i.e., the internal force to defend one's attitudes) is more predictive of resistance than traditional threat measures that focus on apprehension. Future studies should replicate our study with measuring both apprehensive and motivational threat to better establish the theoretical integrity and functioning of inoculation‐based interventions.

This study has contributed to the literature on problematic online use interventions as it is one of the first attempts to introduce and design an attitudinal inoculation‐based intervention. The intervention was specifically tailored to build resistance against persuasion in social media. Although the findings are preliminary, they provide insight into the potential of such interventions, opening up avenues for future directions. Although early research on inoculation viewed it primarily as a preventive approach,^54^ recent studies have suggested that inoculation interventions can also have a treatment effect.^58^ In this vein, future research might delve into the impact of this approach on individuals with severe PSMU, examining both preventive and therapeutic potentials. Moreover, continued investigation of the proposed design could potentially pave the way for a cost‐effective intervention to address problematic online behavior. By utilizing the materials from this study, educators and policymakers could offer training or workshops to help students resist the persuasive nature of technology from a young age. Furthermore, it has the potential for real‐time intervention that designers could employ. Drawing from the work of Kiyak et al.,^116^ which applied informative as well as distractive and cognitive tasks for online gambling, their research showed that such technology‐based interventions can drive positive change.

Our findings showed a potential for inoculation‐based intervention to combat PSMU. Future research may replicate the study with a larger sample size while incorporating an elicited threat measure and varying attitude levels and confidence levels. Further investigations might also benefit from assessing the effectiveness of the intervention components, such as the booster session. Furthermore, as emphasized by Banas and Rains,^70^ there is a need to explore the effects of inoculation intervention decay, which is important in understanding their long‐term implications. Future investigations might examine our study further by evaluating the rate of decay of the inoculation intervention effect as well as the booster session. Such an evaluation would yield important insights into when booster sessions may be required. Additionally, future studies might examine other potential factors that could impact the process of inoculation, such as demographics and need for cognition (NFC). For example, previous studies have found that individuals high in NFC were more likely to elaborate on inoculation messages^90^ and were more resistant to attitude change than those low in NFC.^117^

## CONCLUSION

In addressing PSMU, existing interventions either lack empirical support or are typically cost‐intensive. The widely recognized attitudinal inoculation, while successfully applied in areas like counteracting fake news and smoking,^25^, ^28^ remains an unexplored avenue for PSMU. This study introduced and evaluated an innovative approach grounded in the attitudinal inoculation theory to boost resistance toward problematic online behavior, specifically targeting the problematic use of social media. Overall, the findings demonstrated that an attitudinal inoculation‐based intervention may offer a potential cost‐effective method to combat problematic online behavior. Nevertheless, as these findings are preliminary, they should be interpreted within the context of the study's limitations.

## AUTHOR CONTRIBUTIONS

Sameha Alshakhsi conceptualized and designed the study, curated and analyzed the data, prepared figures and tables, and wrote the first draft. Dena Al‐Thani participated in the study conceptualization and design, reviewed the drafts of the article, and provided feedback. Niko Männikkö reviewed the drafts of the article and provided feedback. Raian Ali participated in the study conceptualization and design, guided the data curation and analysis, reviewed the drafts of the article, and provided feedback. All authors approved the final version of the article.

## CONFLICT OF INTEREST STATEMENT

The authors declare no conflicts of interest.

## Data Availability

The dataset associated with this work is uploaded alongside the Supporting Information for this article at: https://osf.io/69am3/.
